# Aromatic hydrocarbon data in sediments from Port Valdez, Alaska

**DOI:** 10.1016/j.dib.2021.107486

**Published:** 2021-10-17

**Authors:** David G. Shaw, Arny L. Blanchard

**Affiliations:** College of Fisheries and Ocean Sciences, University of Alaska Fairbanks, United States

**Keywords:** Port Valdez, Alaska, Long-term monitoring, Aromatic hydrocarbons

## Abstract

Polycyclic aromatic hydrocarbons (PAH) were measured in sediments from 14 locations in Port Valdez, Alaska in an effort to understand changes associated with the operation of a marine terminal where crude oil delivered by pipeline was transferred to tankers for marine shipment. Samples of surficial sediment were collected annually from 1989 through 2019 at water depths of roughly 30 to 240 m by haps corer of Van Veen grab. PAH concentrations were determined by flame-ionization gas chromatography from 1989 to 2002 and by single ion monitoring gas chromatography-mass spectrometry from 2003 to 2019. Geographic coordinates and environmental variables (water depth, total organic carbon, and sediment grain-size) were also determined. The data are useful for comparisons to trend data elsewhere as well as the investigation of measurement uncertainty in chemical measurements.

## Specifications Table

**Table:** 

Subject	Environmental Science
Specific subject area	Environmental Chemistry, Environmental Management, Monitoring, Policy and Law, and Water quality
Type of data	Table, Graph, Figure
How data were acquired	PAH concentrations were measured using Soxhelt extraction, fractionation by column chromatography and quantification by flame-ionization gas chromatography from 1989 to 2002 and by single ion monitoring gas chromatography-mass spectrometry from 2003 to 2019. See Blanchard and Shaw [1] for further details and quality assurance and quality control procedures.
Data format	Formatted raw data (Table A-1-A3 in Appendix A); Processed and analysed data (Fig. 1).
Parameters for data collection	Samples were collected adjacent to and remote from a point discharge in Port Valdez, Alaska (Table 2). Sampling locations were selected for comparison between stations near the outfall for a ballast water treatment plant and those remote from the discharge. Samples were collected from roughly 30m to 240 m water depth in largely muddy sediments. Sampling of sediments for PAH measurements was conducted each year from 1989 to 2019 with 14 locations sampled in 2019.
Description of data collection	Sediment samples were collected using a Haps from 1989 to 2014 and a van Veen grab from 2014 to 2019. The number of replicate samples analyzed varied from one to three. A sub-sample from the top of each sample was collected for determination of hydrocarbons, total organic carbon (TOC: % dry weight of sediment determined by combustion), and percent sand (the fraction >0.63mm and <2.0 mm by dry weight). See Blanchard and Shaw [1] for further details of the data collection methods.
Data source location	Research conducted by the College of Fisheries and Ocean Sciences, University of Alaska Fairbanks in Port Valdez, Alaska, USA (61° 6′ N, 146° 30′ W)
Data accessibility	With the article
Related research article	A.L. Blanchard, D.G. Shaw, Multivariate analysis of polycyclic aromatic hydrocarbons in sediments of Port Valdez, Alaska, 1989–2019, Mar. Poll. Bull. In Submission. https://doi.org/10.1016/j.marpolbul.2021.112906

## Value of the Data

• These data are a component of long-term environmental sampling program in Alaska. We anticipate that these data will be most useful for those interested in long-term monitoring of PAH concentrations in nearshore marine sediments as the data provide a record of change and variability in an area with anthropogenic impacts. In addition, the data have already been used to explore the role of non-uniform surrogate recovery in measurement uncertainty estimation and other uses are likely to be found.
• Environmental scientists, managers, and policy makers will benefit from these data as they provide an unusually long (31 years) record of PAH concentrations in sediments around a petroleum shipping terminal in an otherwise relatively non-industrialized area.
• These data will provide a basis for others to evaluate uncertainty in field measurements [2] as well as for comparisons with other environmental research programs using aromatic hydrocarbon data.

## 1 Data Description

Integrated multidisciplinary environmental research has been conducted in the marine environment of Port Valdez, Alaska since 1971 [3]. Sediment chemistry has been a part of the Port Valdez Environmental Studies Program since its inception with the goal of documenting concentrations of hydrocarbons in sediments, describing spatial and temporal variability, and determining long-term trends as related to permitted discharges from the Valdez marine oil terminal. Continuing research has focused on long-term change in sediment conditions [1] and uncertainty surrounding concentration estimates [4].

**Fig. 1:**
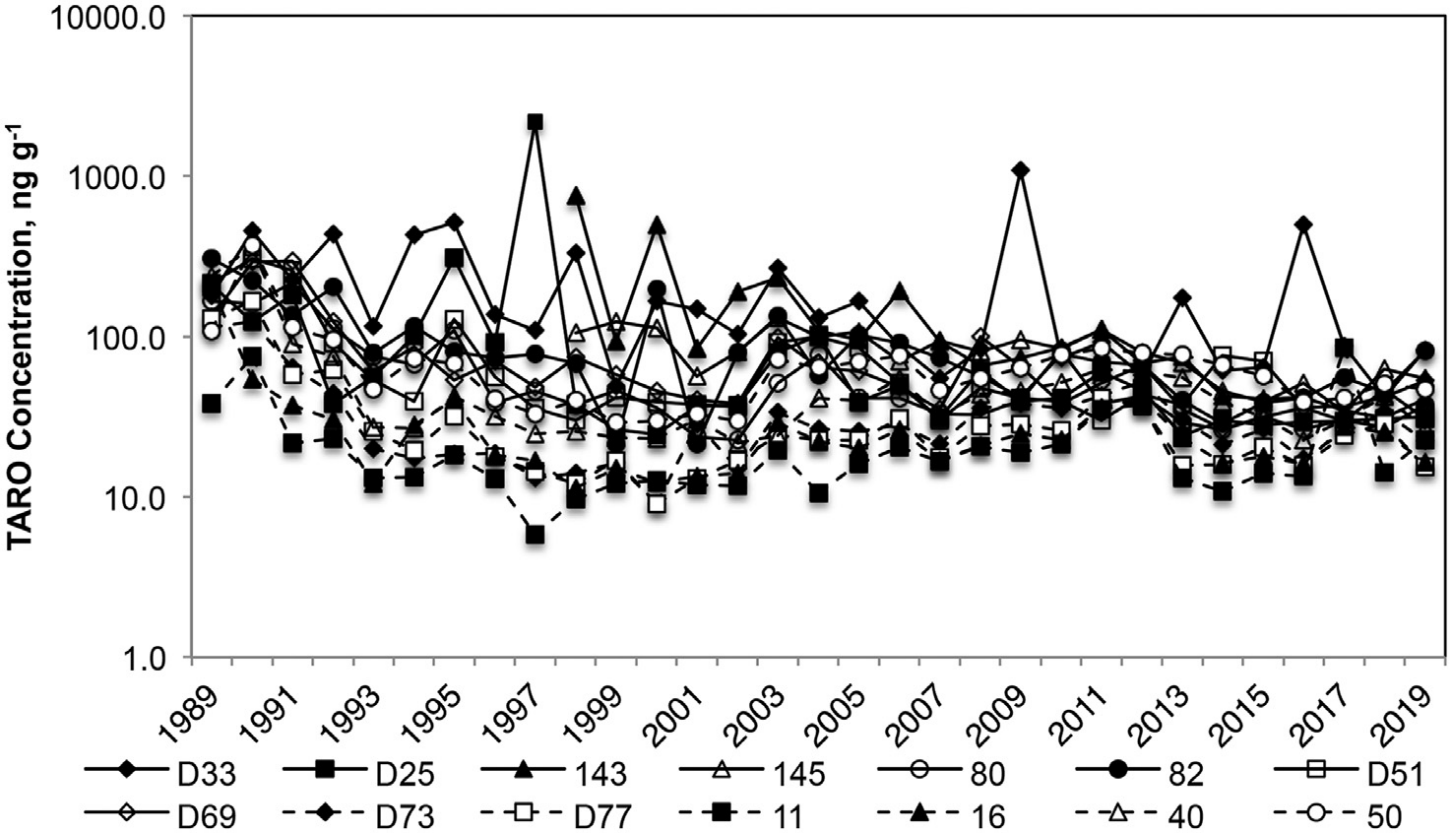
Concentrations of total aromatic hydrocarbons (TARO: Table 1) at 14 sediment locations in Port Valdez, Alaska. Note that the concentration scale is logarithmic.

Fourteen locations were sampled in Port Valdez, Alaska, 1989–2019 [1] as part of the multi-disciplinary research program [5,6]. Geographic coordinates of sampling locations and target water depths of the sampling locations are given in Table 1. Hydrocarbon concentrations have declined over time (Fig. 1) and exhibit significant spatial and temporal variability. Summary measures calculated from the data include measures of total aromatics and low and high molecular weight aromatics; the association of individual analytes with summary measures are detailed in Table 2.

**Table 1:** Intended locations (NAD 1983) and water depth (midpoint and expected ranges) of stations from which sediments were sampled for this project.

Group	Station	Depth, m	Latitude, N	Longitude, W
Shallow	D33	64 (48-80)	61 05 26.5867	146 23 02.7447
	D25	71 (61-81)	61 05 27.7023	146 23 16.7320
	80	78 (65-90)	61 05 29.9160	146 24 22.2402
	82	52 (37-67)	61 05 26.7540	146 22 22.8987
	D51	83 (65-100)	61 05 24.1538	146 23 30.3457
	D69	80 (59-100)	61 05 23.9847	146 23 46.5949
	143	70 (60-80)	61 05 23.4000	146 23 19.2000
	145	75 (65-85)	61 05 25.2000	146 23 12.0000
Deep	D73	220 (195-245)	61 05 38.8463	146 23 24.1898
	D77	237 (235-238)	61 05 45.0718	146 22 41.7289
	11	224 (197-251)	61 06 19.1174	-146 20 07.2810
	16	233 (225-240)	61 05 52.1129	-146 21 55.2776
	40	242 (230-253)	61 06 19.1158	146 28 49.3046
	50	247 (243-250)	61 06 19.1082	146 35 49.3106

**Table 2:** Analytes used in this study and their relationship to summary measures.

Compound	TARO	HARO	LARO	LPAH	HPAH	TPAH
1,6,7-Trimethylnaphthalene				X		X
1-Methylnaphthalene	X		X	X		X
1-Methylphenanthrene	X		X	X		X
2,6-Dimethylnaphthalene	X		X	X		X
2-Methylnaphthalene	X		X	X		X
Acenaphthene	X		X	X		X
Acenaphthylene				X		X
Anthracene	X		X	X		X
Benz[a]anthracene	X	X			X	X
Benzo[a]pyrene	X	X			X	X
Benzo[b]fluoranthene					X	X
Benzo[e]pyrene	X	X			X	X
Benzo[g,h,i]perylene					X	X
Benzo[k]fluoranthene					X	X
Benzothiophene				X		X
Biphenyl	X		X	X		X
Carbazole				X		X
Chrysene	X	X			X	X
Dibenzo[a,h]anthracene	X	X			X	X
Dibenzofuran				X		X
Dibenzothiophene				X		X
Fluoranthene	X	X			X	X
Fluorene	X		X	X		X
Indeno[1,2,3-c,d]pyrene					X	X
Naphthalene	X		X	X		X
Naphthobenzothiophene					X	X
Perylene	X	X			X	X
Phenanthrene	X		X	X		X
Pyrene	X	X			X	X

Aromatic hydrocarbon concentrations from Port Valdez, Alaska, 1989–2019 are provided in Supplementary Table 1. The data included are the year of sampling, station, replicate, the geographic coordinates (Latitude and Longitude, NAD83), environmental variables, and polycyclic aromatic hydrocarbon (PAH) concentrations (uncorrected and for 2003–2019, surrogate-corrected concentrations). Intended sampling locations and target water depths were included for 1989–2002 whereas GPS locations of sampling points and replicate water depths are available for 2003. From 1989–2002, sediment grain-size fractions are available for percent gravel, sand and mud from a single replicate for each station and that value is used as representative for all replicates. From 2003 to 2019, grain-size data are available for each replicate.

Surrogates and recoveries for 1989–2002 are included in Supplementary Table 2. These surrogate recoveries were used for QA/QC control but were not used to correct PAH data.

The surrogates used for individual analytes, 2003–2019 are listed in Supplementary Table 3.

Surrogate recoveries for 2003–2019 are included in Supplementary Table 4. The recoveries were used to calculated surrogate-corrected concentrations by dividing uncorrected analyte concentrations by their surrogate recoveries.

## 2 Experimental Design, Materials and Methods

Sampling of sediments for PAH measurements was conducted each year from 1989 to 2019 with 14 locations sampled in 2019 in Port Valdez, Alaska (Table 1). Sediment samples were collected using a Haps corer [7] from 1989 to 2014. Sampling with the Haps corer was difficult in the compact sediments resulting in partially filled cores, disturbed cores, and extended sampling efforts at some locations. Thus, a van Veen grab [8] was used from 2014 to 2019. Long-term sampling stations include six deep (11, 16, 40, 50, D73, and D77) and eight shallow stations (80, 82, 143, 145, D25, D33, D51, and D69). Replication varies from one to three replicates as called for by the discharge permit for which this monitoring was conducted and all unequal replication arises from permitting decisions and not missing samples. Prior to use the sampling equipment was cleaned by washing with Liquinox (TM) detergent followed by rinsing with tap water and deionized water. Samples were visually examined and rejected when they were underfilled, there was evidence of over-filling of the sample or substantial wash-out was noted. See Blanchard and Shaw [1] for further details.

A sub-sample from the top of each sample was collected for determination of hydrocarbons, total organic carbon (TOC: % dry weight of sediments determined by combustion), and gravel (the fraction >2.0 mm by dry weight), percent sand (>0.63mm and <2.0 mm), and mud (<0.63mm). Water depth (m) and geographic coordinates (Latitude, Longitude; NAD 1983) were also determined. Beginning in 1998, station locations were determined by DGPS and available for each sampling point (replicate). Station locations are determined using a GARMIN GPSmap 76CSx with an external antenna. Prior to 1998, only general (intended) geographic locations are available. From 1989 to 2002, grain-size analysis was conducted on single replicate samples for each station and those values were used for all replicates for a station.

During the period 1989 to 2002, PAH concentrations were measured by the methods of MacLeod et al. [9] and Krahn et al. [10] using Soxhelt extraction of sediment samples, fractionation by column chromatography, and quantification by flame-ionization gas chromatography. Since 2003, laboratory analyses were performed by TDI-Brooks International, Inc, College Station, Texas using methods based on USEPA Methods 3500C and 8100 [11,12] using single ion monitoring gas chromatography-mass spectrometry (SIM-GC/MS). Methodological changes have balanced the need to improve the quality of the data with the need to protect the long-term integrity of the data. Quality assurance and quality control (QA/QC) procedures were performed according to the laboratory's standard operating procedures. Quality control limits were set for each QA/QC element prior to these analyses and variances from these limits, if any, were reported. Analysis of standard reference materials such as NIST SRM (1944 New York / New Jersey Waterway Sediment) indicated no systematic difference between results obtained by the two methods.

The major change in analytical technique in 2003 was the replacement of identification and quantification of PAH by flame-ionization gas chromatography with gas-chromatography mass spectrometry which allowed the resolution of constituents with similar or overlapping retention times and method refinements led to decreases in detection limits over the course of the work. Detection limits for individual PAH were in the range 1 to 10 ng g^−1^ in 1989 and declined to 0.1 to 1 ng g^−1^ in 2019. A suite of 18 analytes (the target compounds for measurement) were measured in 1989–2002 and 33 analytes since 2003 (including the prior 18 analytes) (Table 2). The concentrations measured since 2003 are reported both uncorrected and surrogate corrected while the data from 1989-2002 are reported uncorrected only. Concentrations (mass fraction, ng g^−1^) of 18 PAHs have been measured over the whole study period 1989–2019 and can be summarized as total aromatics (TARO), total high-weight aromatics (HARO), and total low-weight aromatics (LARO) (Table 2). Similar calculations can be made for surrogate-corrected data 2003–2019 as TPAH, HPAH, and LPAH. Although hydrocarbon measurements were made prior to 1989, that work focused on alkanes and individual PAH were not quantified.

The improvements in analytic techniques and reporting standards over the time period led to increased data quality in the later years. In addition, permit compliance requirements changed over the collection period leading to differences in the suite of stations sampled and the number of replicate samples analyzed between years. Nevertheless, this dataset has value as an unusually long series of measurements made on an annual basis.

## Ethics Statement

This work does not contain any studies with humans, animals from protected areas, or endangered animals.  The authors declare that they have followed the general ethics rules for scientific research and publishing.

## CRediT authorship contribution statement

**David G. Shaw:** Conceptualization, Methodology, Supervision, Project administration, Validation, Writing – original draft. **Arny L. Blanchard:** Conceptualization, Methodology, Formal analysis, Data curation, Writing – original draft, Project administration.

## Declaration of Competing Interest

The authors declare that they have no known competing financial interests or personal relationships which have or could be perceived to have influenced the work reported in this article.

## Appendix Supplementary Materials

Image, application 1

Image, application 2

Image, application 3

Image, application 4
